# Image-Based Profiling of Synaptic Connectivity in Primary Neuronal Cell Culture

**DOI:** 10.3389/fnins.2018.00389

**Published:** 2018-06-26

**Authors:** Peter Verstraelen, Michiel Van Dyck, Marlies Verschuuren, Nachiket D. Kashikar, Rony Nuydens, Jean-Pierre Timmermans, Winnok H. De Vos

**Affiliations:** ^1^Laboratory of Cell Biology and Histology, Department of Veterinary Sciences, University of Antwerp, Antwerp, Belgium; ^2^Janssen Research and Development, Janssen Pharmaceutica N.V., Beerse, Belgium; ^3^Cell Systems and Imaging, Department of Molecular Biotechnology, Ghent University, Ghent, Belgium

**Keywords:** primary neuronal culture, synapse, dendritic spine, image analysis, morphofunctional connectivity, fluorescent labeling, high-content screening

## Abstract

Neurological disorders display a broad spectrum of clinical manifestations. Yet, at the cellular level, virtually all these diseases converge into a common phenotype of dysregulated synaptic connectivity. In dementia, synapse dysfunction precedes neurodegeneration and cognitive impairment by several years, making the synapse a crucial entry point for the development of diagnostic and therapeutic strategies. Whereas high-resolution imaging and biochemical fractionations yield detailed insight into the molecular composition of the synapse, standardized assays are required to quickly gauge synaptic connectivity across large populations of cells under a variety of experimental conditions. Such screening capabilities have now become widely accessible with the advent of high-throughput, high-content microscopy. In this review, we discuss how microscopy-based approaches can be used to extract quantitative information about synaptic connectivity in primary neurons with deep coverage. We elaborate on microscopic readouts that may serve as a proxy for morphofunctional connectivity and we critically analyze their merits and limitations. Finally, we allude to the potential of alternative culture paradigms and integrative approaches to enable comprehensive profiling of synaptic connectivity.

## Introduction

The term ‘synapse’ was first coined in 1897 for the anatomical location where two neurons interact (Foster and Sherrington, 1897). Since then, new developments in electrophysiology, microscopy, and biochemistry have ensured that the biochemical aspects of chemical synapses are now well characterized (O’Rourke et al., 2012). In a very reductionist view, the synapse can be regarded as a polarized unit, which consists of a presynaptic compartment at one neuron and a postsynaptic compartment at a second, target neuron, which are separated by a 10–20 nm wide extracellular space, the synaptic cleft. Inhibitory synapses are located on the dendritic shaft, while at excitatory synapses, the postsynaptic compartment often forms a micron-sized protrusion that is known as a dendritic spine. At the presynaptic side, voltage-gated calcium influx induces neurotransmitter release from vesicles into the synaptic cleft. In case of an excitatory synapse, subsequent activation of neurotransmitter receptors at the postsynaptic side induces depolarization in the target neuron, which – when summed across multiple synapses in time – can lead to the generation of a new action potential. It has also become clear that synapses are dynamic structures, and that activity-dependent changes in synaptic strength underlie learning and memory (Whitlock et al., 2006). Learning-induced adaptations leave a multi-synaptic memory trace of functionally connected neurons within the network that facilitates rapid memory retrieval upon future experience (Mongillo et al., 2017). The strength of individual synapses within this memory trace is determined by their presynaptic release probability, as well as by postsynaptic mechanisms such as neurotransmitter receptor abundance or dendritic spine morphology. As such, functional synapse changes often coincide with structural adaptations that can be used as morphological correlates of synaptic strength. The overall synaptic strength of the neuronal network – rather than the strength of a single synapse – is referred to as ‘synaptic connectivity’ (Chklovskii, 2004; Litwin-Kumar et al., 2017).

It is exactly this capacity to change and maintain synaptic connectivity (i.e., synaptic plasticity) that becomes compromised in several neurological disorders. The majority of genetic risk factors identified in schizophrenia converge onto pathways that regulate synaptic plasticity, such as the *N*-methyl-D-aspartate receptor (NMDA-R) signaling complex, activity-regulated cytoskeletal interactors, and voltage-gated calcium channels (Hall et al., 2015). In Alzheimer’s disease, soluble β-amyloid and hyperphosphorylated Tau oligomers cause synaptotoxic effects that are considered to be responsible for typical dementia symptoms such as memory loss and behavioral deterioration (Spires-Jones and Hyman, 2014). Although the exact mechanisms that underlie these synaptic defects are not yet fully resolved, it is conceivable that a more synapse-oriented approach may expedite the development of disease-modifying treatments. This demands apt interrogation paradigms for quantifying synaptic connectivity with high sensitivity.

With more than 100 billion neurons, even more glial cells, and trillions of synapses (Herculano-Houzel, 2009) organized into a dense, three-dimensional network, the brain does not offer a facile access route to quickly measure synaptic connectivity. In addition, intact brains are not easily amenable to multiplexing. Hence, neuroscientists often revert to simplified *in vitro* models such as primary neuronal cultures (e.g., hippocampal or cortical) extracted from rodent embryos. These cultures recapitulate several features of *in vivo* neuronal networks including outgrowth of dendrites and axons, formation of synapses between pre- and postsynaptic partners, and presence of dendritic spines. Furthermore, they display synchronous NMDA-R-mediated activity, as measured by calcium imaging at the level of the neuronal network (Verstraelen et al., 2014) and of the individual synapse (Jackson and Burrone, 2016). Hence, such a model can be used to assess the molecular mechanisms that govern synaptic connectivity with high resolution and throughput.

**FIGURE 1:**
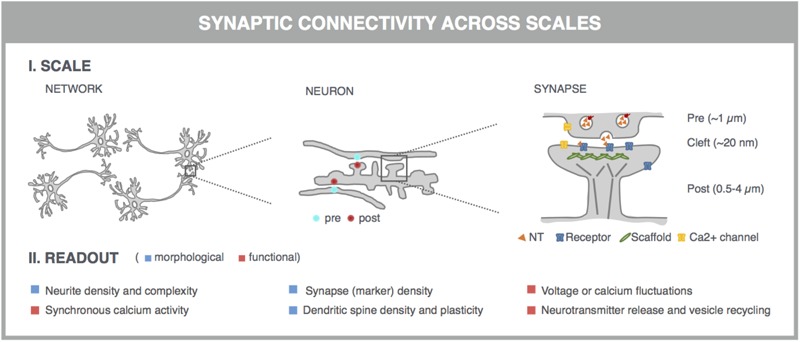
Microscopy-based readouts for synaptic connectivity at different scales. Synaptic connectivity can be investigated on different scales (network, individual neurons and synapses), which come with characteristic morphological and functional readouts. Clues into synaptic connectivity are already inferred from the general network architecture. Neurite density and complexity (e.g., length and branching points) inform about the general health and connectivity of the neuronal network. The synchronicity of spontaneous neuronal activity, measured across neuronal cell bodies, is used as a readout for functional connectivity. The number of synaptic connections serves as a direct readout of neuronal connectivity and can be quantified after fluorescent labeling of synapse marker proteins such as presynaptic vesicle proteins (e.g., synaptophysin) and postsynaptic neurotransmitter (NT) receptors (e.g., AMPA-R) or scaffold proteins (e.g., PSD-95). This assay can be refined by measuring the colocalization of pre- and postsynaptic markers, which can be excitatory or inhibitory. Excitatory synapses are often localized on dendritic spines, actin-rich protrusions from the dendritic shaft whose density and morphology correlate with synaptic strength. Synaptic transmission can be directly visualized in dynamic assays, using specific fluorescent reporters. At the presynaptic side, synaptic vesicle acidification, calcium influx, or membrane recycling can be probed. Release of neurotransmitters such as glutamate (orange triangles) into the synaptic cleft can be visualized, as well as the postsynaptic depolarization that they induce via calcium or voltage imaging. Some of these markers can be targeted to pre- or postsynaptic compartments by fusing them to the aforementioned synaptic markers.

**FIGURE 2:**
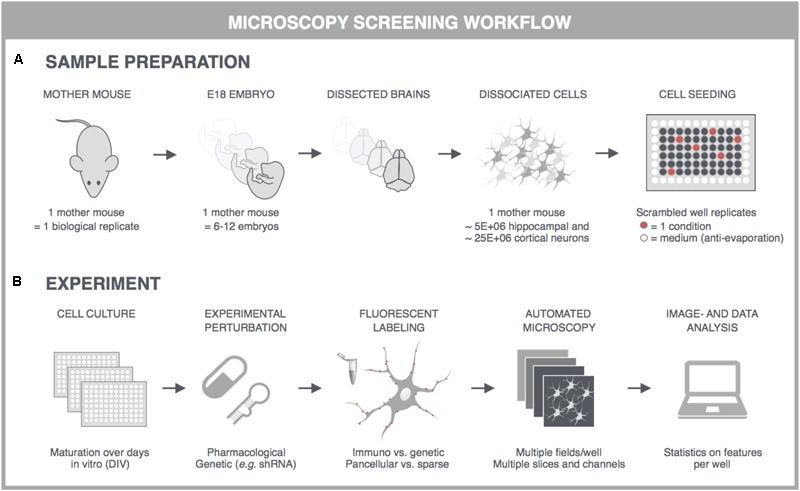
General principles for high-content screening with primary neuronal cultures. Although the sample preparation is more tedious and variable, primary cultures offer a level of synaptic connectivity that cannot be matched by immortal cell lines. The following steps are typically followed in synapse, spine or functional screens. **(A)** One dam is regarded as one biological replicate, and embryos, typically E17-18, of the same dam are pooled to obtain sufficiently large suspensions of cortical or hippocampal cells. Cells are seeded into multiwell plates of which the outer wells are filled with sterile medium, and the well replicates scrambled to avoid edge effects. **(B)** In high-content experiments, neuronal cultures are dosed and fluorescently labeled using an automated liquid handling system (genetic labeling is usually done before perturbation whereas immunostaining after). Images are captured on an automated microscope which is equipped to allow rapid acquisition, e.g., by employing multiple sensitive cameras with large fields-of-view for parallelization of fluorescence channels. Since primary neuronal cultures can show a heterogeneous distribution, multiple fields are captured per well. These fields are analyzed with high-content image analysis scripts and the resulting data is presented per well. Finally, statistics, data mining and visualization aid the interpretation, after which a secondary screen or low-throughput confirmation experiments can be conceived. This figure was adapted from Verstraelen et al. (2017) with permission.

With the advent of automated, highly multiplexed, high-content microscopy, it has now become possible to derive information on (synaptic) connectivity in primary neuronal networks with deep coverage (**Figure 1**). In this context, both morphological and functional readouts can be extracted on multiple scales. At the level of the entire neuronal network, it is possible to infer, to some extent, how well connected individual neurons are by quantifying the density of neurites (axons and dendrites) or by measuring the synchronicity of spontaneous electrical activity in neuronal cell bodies. As a more direct morphological readout, the number of synaptic connections can be quantified after fluorescent labeling of synapse markers. When combining a pre- and postsynaptic marker, the overlap of both can be used as a proxy for mature synapses. An alternative morphological readout focuses on the dendritic spines, since both the number and morphology have been correlated with the state of connectivity (Moyer et al., 2015; Herms and Dorostkar, 2016; Martínez-Cerdeño, 2017). Lastly, synaptic transmission can be measured in functional assays that focus on presynaptic vesicle turnover, neurotransmitter release, or postsynaptic responses. Despite different technicalities, all these readouts can in principle be obtained in a high-throughput setting that relies on a general workflow depicted in **Figure 2**. In brief, such a workflow relies on parallel cultures of cortical or hippocampal neurons prepared from brains of rodent embryos (typically at embryonic day 18, E18). These initially spherical cells spontaneously grow axons and dendrites, and establish synaptic contacts over days to form morphologically and functionally interconnected networks. After a sufficiently long culture period (at least 7 days *in vitro*, DIV7), cultivated neuronal networks are exposed to pharmacological or genetic perturbations that potentially modulate connectivity during maturation or thereafter. Fluorescent labeling of the above-mentioned correlates of synaptic connectivity (synapses, spines, and transmission reporters) is monitored by automated microscopy (enabling acquisition of large image data sets per well) and combined with high-content image analysis (enabling extraction of large information content per image) to extract quantitative data with statistical power.

## Quantification of Synapses Using Markers

### Visualizing Individual Synapses

**FIGURE 3:**
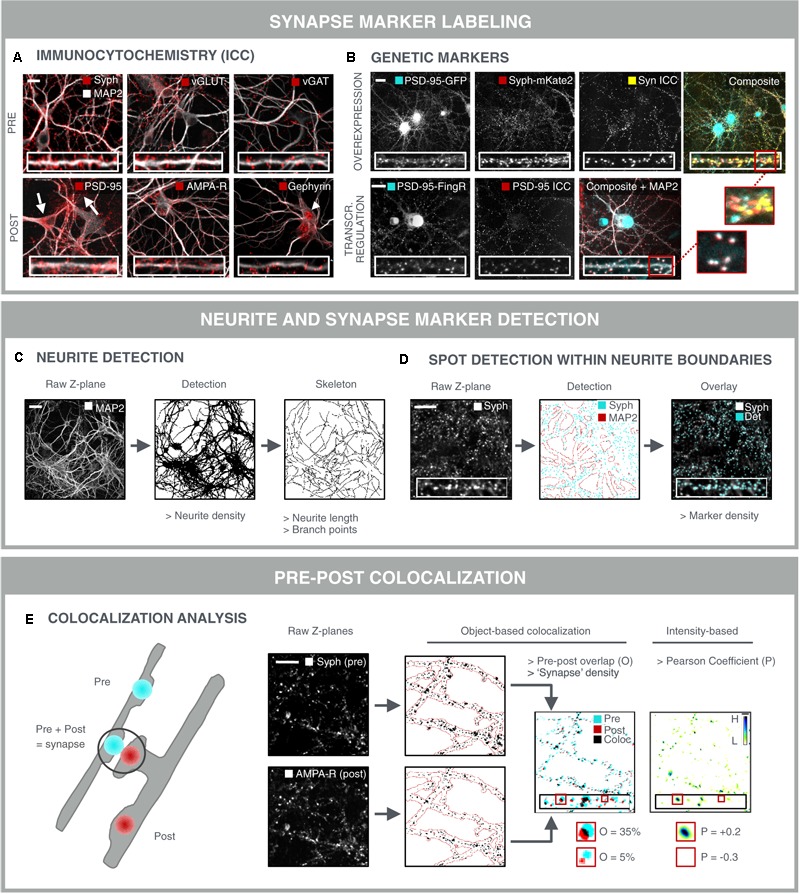
Synapse marker quantification as morphological readout for connectivity. **(A)** Immunocytochemical labeling (ICC) of pre- and postsynaptic markers in primary hippocampal neurons to label all [synaptophysin (Syph)], excitatory (vGLUT, PSD-95, and AMPA-R) or inhibitory (vGAT, gephyrin) synapse compartments. Some antibodies give rise to non-specific staining in neuronal cell bodies and astrocytes (PSD-95; arrows) or in neuronal nuclei (gephyrin; arrowhead), underlining the need for thorough antibody validation. Scale bar: 20 μm. **(B)** Genetic labeling of synaptic markers allows for temporal follow-up. Constitutive overexpression of PSD-95-GFP and synaptophysin-mKate2 (Syph-mKate2) gives rise to overexpression artifacts such as overfilling of the neuronal soma and dendrites by PSD-95-GFP, and a high number of synaptophysin-mKate2 puncta that do not colocalize with ICC of another presynaptic protein, synapsin (Syn). Targeting of GFP toward endogenous PSD-95 using FingR technology (Gross et al., 2013) results in good colocalization of PSD-95-FingR and ICC, making this approach more attractive for synapse screening than constitutive overexpression of fusion constructs. Scale bars: 20 μm. **(C)** MAP2-stained neurites can be analyzed for area fraction, but also skeletonized to determine the length and number of branching points. Scale bar: 50 μm. **(D)** Synaptic marker spots are segmented within the boundaries of the neurite mask to calculate synaptic marker density. An overlay of the raw image and the detected spots shows accurate spot detection. Scale bar: 20 μm. **(E)** To avoid detection of immature synapses, extrasynaptic and a specific staining, the apparent colocalization of pre- and postsynaptic markers can be evaluated as a proxy for mature synapses. Pre- and postsynaptic images of corresponding Z-planes are considered to avoid overdetection of mature synapses from different axial positions. After segmentation, pre- and postsynapse masks are combined and analyzed for colocalization, yielding parameters such as % overlap (O) or synapse density. A mature synapse can be defined by an arbitrary cut-off, e.g., 30% overlap between post/pre or pre/post. Alternatively, an intensity-based Pearson coefficient (P) can be calculated for the pre- and postsynaptic images. This method is independent of spot segmentation. For visual representation, the ‘product of the differences from the mean’ (PDM) is shown (H, high colocalization; L, low). Scale bar: 20 μm.

The most direct approach for investigating synaptic connectivity in primary neuronal culture, is to visualize and count the number of synapses. A variety of antibodies has been developed to label synapse-specific components in pre- and postsynaptic compartments (Biederer and Scheiffele, 2007) (**Figure 3A**). At the presynaptic side, such markers are mostly located on neurotransmitter vesicles. Synaptophysin for instance, is present on virtually all presynaptic vesicles but, despite its abundance, its role is not entirely clear. Knockout mice show normal synaptic transmission (McMahon et al., 1996), but more recently, synaptophysin was found to regulate the kinetics of synaptic vesicle endocytosis (Kwon and Chapman, 2011). Next to this pan-synaptic marker, it is also possible to differentiate between excitatory and inhibitory terminals. For example, vGLUT1 and vGAT are commonly used markers for excitatory and inhibitory terminals, respectively. vGLUT1 catalyzes glutamate transport (Wojcik et al., 2004), whereas vGAT loads the inhibitory neurotransmitters GABA and glycine into pre-synaptic vesicles (Chaudhry et al., 1998). Unfortunately, there are no generic post-synaptic markers. It is possible to differentiate excitatory from inhibitory synapses at the postsynaptic side. To this end, both scaffold proteins or neurotransmitter receptors can be targeted. Postsynaptic density protein 95 (PSD-95) is a structural scaffold protein that anchors glutamate receptors such as α-amino-3-hydroxy-5-methyl-4-isoxazolepropionic acid receptors (AMPA-R) and NMDA-R, and is an important regulator of excitatory synaptic strength (Chen et al., 2011). Likewise, gephyrin anchors GABA- and glycine receptors, while it also interacts with cytoskeletal components to regulate inhibitory synapse plasticity (Tyagarajan and Fritschy, 2014). Neurotransmitter receptors are transmembrane proteins that can be labeled with antibodies targeting an extracellular epitope. By omitting the permeabilization step during immunolabeling, the membrane-bound receptors are selectively labeled, while intracellular and hence non-functional receptors remain undetected. Still, a considerable fraction of membrane-associated receptors is extra-synaptic, thereby diluting the specific synaptic signal (Brickley and Mody, 2012; Schmidt-Salzmann et al., 2014). It is also important to take into account that the choice of the synapse marker determines the nature of the assay. Although individual PSD-95 proteins can leave and re-enter the postsynaptic density in a matter of hours, the postsynaptic density as a whole persists for several days (Gray et al., 2006). In contrast, AMPA-R are inserted into or removed from the postsynaptic membrane within minutes after induction of long-term potentiation (LTP) or long-term depression (LTD) (Hayashi et al., 2000; Ehlers et al., 2007; Kessels and Malinow, 2009). As such, AMPA-R labeling offers a means to assess functional differences and the effect of acute treatments, while PSD-95 is likely to respond to chronic treatments and structural changes only.

Although immunolabeling offers the possibility to label a variety of markers at the same time, it suffers from a few drawbacks such as non-specific staining and batch-to-batch variability, and it cannot be used for temporal follow-up of the same samples (longitudinal studies). To overcome these limitations, genetically encoded synapse markers have been developed (Luo et al., 2002; De Paola et al., 2003; Ehlers et al., 2007; Williams et al., 2011) (**Figure 3B**). However, constitutive expression of fluorescent fusion proteins is prone to inducing overexpression artifacts by perturbing endogenous synaptic protein homeostasis (Prelich, 2012). This can result in more and larger synapses, as well as in filling of the neurites with excess fluorescent protein. Therefore, stoichiometric labeling of endogenous proteins is preferred, as proposed for PSD-95 by ‘endogenous labeling via exon duplication’ (ENABLED) (Fortin et al., 2014). Another elegant strategy to minimize overexpression artifacts has been proposed by Gross et al. (2013) in the form of FingRs. FingRs target GFP to the endogenous synapse proteins PSD-95 or gephyrin. FingR expression levels are matched to the levels of the endogenous target using an ingenious transcriptional feedback regulation system, in order to reduce the background fluorescence in the neurites. The authors showed that synapse size, morphology or electrophysiological properties are not altered by FingR expression, as opposed to constitutive overexpression of a fusion protein.

Fluorescent proteins do come with other caveats such as limited spectral flexibility, low signal intensities, and photobleaching. Recently, chemical labeling methods have been conceived, which use ultra-bright synthetic organic dyes that can specifically attach to genetically encoded protein tags such as SNAP, CLIP, HALO, and TMP (Lavis, 2017). Through fluorescence microscopic studies, it was shown that this ultrafast and even labeling method works when applied to Drosophila and mouse circuits in the brain, when investigating individual neuronal types (Kohl et al., 2014; Yang et al., 2014; Sutcliffe et al., 2017). Further iterations of this technology to label proteins of choice and ever-improving fluorophores (Grimm et al., 2017) should pave the way for rapid and more efficient labeling of synaptic circuits.

Synapse images are typically acquired on a confocal fluorescence microscope since such a setup allows visualization of at least four channels in optical sections. Given the small size of synapses, high magnification, high NA lenses are a must. This usually implies the use of immersion media such as water. For high-content screening, high-resolution imaging is fully automated via robotic sample mounting, an automated microscope stage and autofocus system, and automated immersion medium replenishment. The acquisition speed is enhanced by using a high-intensity light source, a spinning disk rather than a scanning confocal setup, and one or more sensitive cameras (to parallelize the acquisition of different fluorescence channels).

### Quantification of Synapse Density

After labeling and image acquisition, the number of synapses should be quantified in a robust manner. Typically, a synapse marker is combined with a neurite marker, e.g., MAP2, to allow facile quantification of the synapse density, i.e., the number of synaptic spots per dendrite length or surface area (**Figures 3C,D**). Skeletonization is used to measure length and branching points (complexity), while the neurite density is obtained by (adaptive) thresholding the image after preprocessing (Pani et al., 2014). The former provides a more detailed view, yet it is much more prone to underdetection in dense regions. Synaptic spots can be detected within the boundaries of the neurite mask to avoid detection of non-specific spots (e.g., from non-neuronal cells), after which spot number, intensity and area can be quantified (**Figure 3D**). A variety of efficient spot detection methods are available (Marr and Hildreth, 1980), and several image analysis packages have been developed specifically for synapse marker detection, such as SynPAnal (Danielson and Lee, 2014), SynD (Schmitz et al., 2011) and the Puncta Analyzer plugin for ImageJ freeware (Ippolito and Eroglu, 2010).

Detection of single synapse markers is extensively reported in literature (Critchlow et al., 2006; Cai et al., 2016; Hui et al., 2016), but the sensitivity of such an assay is limited due to the labeling of immature, non-functional synapses, intracellular (trafficking) proteins, or extrasynaptic markers (cfr. above). Furthermore, there is always some degree of a specific antibody labeling or mistargeted genetic label (Fritschy, 2008; Quattrocchio et al., 2013). To accommodate for these drawbacks, a dual labeling strategy is preferred, in which the (partial) overlap of a pre- and postsynaptic marker serves as a more reliable readout for true synapse density (**Figure 3E**) (Diniz et al., 2012; Nieland et al., 2014; Sellers et al., 2015; Segura et al., 2016). The detection of overlapping pre- and post-synaptic spots is the subject of colocalization analysis. Object-based colocalization relies on the segmentation of spots in both fluorescence channels separately, followed by quantification of their relative overlap (**Figure 3E**) (Ippolito and Eroglu, 2010; Schätzle et al., 2012; van Deijk et al., 2017). Intensity-based colocalization analysis relies on the quantification of the co-variation of the intensities in both channels (Pearson coefficient; **Figure 3E**) (Manders et al., 1993; Adler and Parmryd, 2010; Glebov et al., 2016). The latter is usually calculated across the entire image, but can also be confined to the individual spot regions (at the expense of statistics). It should be noted, however, that the individual markers reside in distinct anatomical compartments and that the apparent colocalization of pre- and postsynaptic markers in reality is an imaging artifact caused by objects that are spaced below the diffraction limit. That is also why precise calibration and benchmarking of the optical imaging setup is imperative. For instance, bleedthrough and chromatic aberrations should be minimized and corrected for (e.g., using synthetic multicolor beads) (Kozubek and Matula, 2000). Some analysis scripts also quantify proximity of pre- and postsynaptic markers, based on the minimal distance of their centers of mass (Schätzle et al., 2012). Although this allows tuning of the proximity criterion, it also raises background due to random proximity, especially in dense cultures.

### Considerations for Accurate Synapse Screening

**FIGURE 4:**
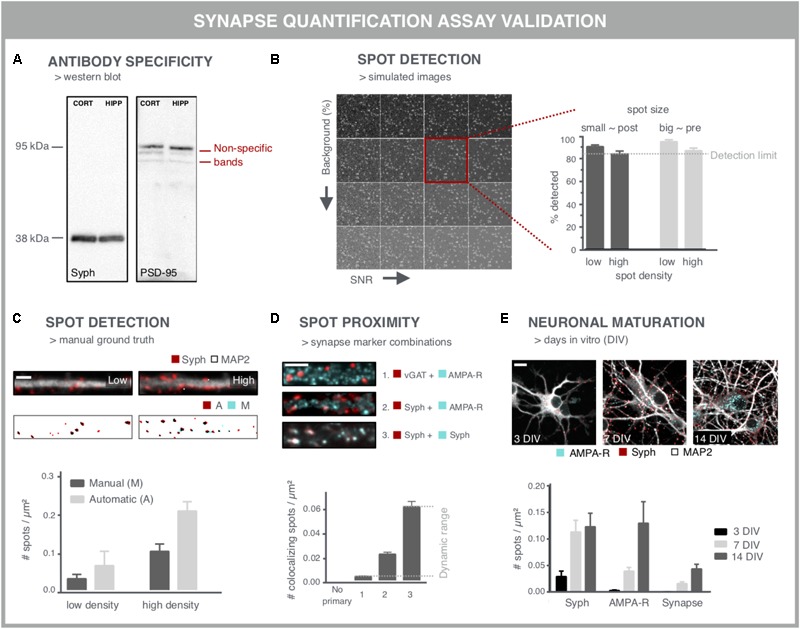
Validation experiments for synapse screening. **(A)** Validation of antibodies via western blot. A Synaptophysin (Syph) antibody shows the expected single band at 38 kDa. Conversely, a PSD-95 antibody shows, next to a bright band at the expected height, additional bands, indicative of non-specific binding (cort/hipp: 14 DIV mouse cortical or hippocampal primary neurons). **(B)** Validation of the spot detection algorithm using simulated images with increasing background (BG) and decreasing signal-to-noise ratio (SNR). Besides background and SNR levels, spot size and density determine the lower detection limit. **(C)** Spot detection algorithms can also be validated by comparison with a ground truth, obtained by manual spot counting in (regions of) real images. The binary images show the detection of spots after manual (M) or automatic (A) detection. Manual spot quantifications may differ (here lower) with those from the spot counting algorithm, but if counts change proportionally in low- and high-density regions, relative comparisons are still possible. Scale bar: 5 μm. **(D)** Validation of colocalization analysis for mature synapses by different marker combinations. The lower detection limit can be pinpointed using an inhibitory presynaptic (e.g., vGAT) and excitatory postsynaptic (e.g., AMPA-R) marker, which only colocalize by coincidence. The upper limit can be determined using two different primary antibodies for the same marker (e.g., synaptophysin). The combination of a pan-presynaptic marker (e.g., synaptophysin) and a specific postsynaptic marker (e.g., AMPA-R) should in turn yield an intermediate level of colocalizing spots, depending on the ratio of excitatory/inhibitory synapses. Scale bar: 5 μm. **(E)** Analysis of cultures at different days *in vitro* (DIV) can be used as a positive control for increasing connectivity, exemplified by an increasing density of pre- and postsynaptic spots. The number of mature synapses also increases, but is markedly lower compared to the pre- and postsynaptic markers alone. Scale bar: 10 μm.

When aiming to use synapse density as a readout for screening purposes, several considerations should be taken into account to ensure that the readout is accurate and reproducible. A first important variable is the specificity of the antibody used for labeling the synaptic markers (**Figure 4A**). Western blots may serve as a quick test to reveal non-specific binding (**Figure 4A**). However, one-to-one comparison between immunocytochemistry and western blot is difficult given the different preparation procedures that alter epitope accessibility. The specificity can be further validated by comparing immunofluorescent staining in control cells and after performing a selective knock-down for the target protein. Another labeling issue that should be taken into account is the fact that neuronal health can interfere with the labeling quality, especially upon genetic labeling. Cellular debris often emits a fluorescent signal that is difficult to distinguish from synaptic spots. Although colocalization analysis of pre- and postsynaptic spots partly accommodates for this problem, the presence of cellular debris is likely to bias a compound screen, especially in the higher (potentially toxic) concentration range. This can, however, be corrected for during data mining as other parameters such as the nuclei number and neurite network density are typically also affected.

A second point of attention is the reliability of the image analysis output. Although synapse marker spots are relatively easy to detect in theory, there are a number of confounding factors that complicate segmentation, such as their abundance and SNR. Another important difficulty relates to the gradual clustering of neurons over time, resulting in aggregated cell bodies and fasciculate neurite bundles in which segmentation of individual synapses becomes problematic. Clustering may be mitigated by growing the cells on differently coated substrates (Sun et al., 2012). One common approach to validate spot detection algorithms are Monte Carlo simulations (De Vos et al., 2010; Harrison, 2010). By analyzing a large set (∼10.000) of synthetically generated images containing randomly distributed spots, the efficiency of the spot detection, or the detection limit, can be defined with statistical accuracy (**Figure 4B**). In order to inform on the sensitivity for the specific image type at hand, simulations should include differently sized spots – presynaptic spots are typically larger than postsynaptic ones – at different densities, and with varying background and SNR (**Figure 4B**).

This artificial approach, while informative, does not exactly mimic real microscopic images, where typically different regions with variable density, background and SNR are found within the same field of view. Therefore, the performance of spot detection efficiency on simulations should be seen as a rough estimate and may differ with the performance on real images (Štěpka et al., 2015). An alternative validation step consists of comparing the spot detection algorithm to a manually annotated ground truth. Comparison with a ground truth has been reported for synapses in neuro-muscular junctions in Drosophila (Nijhof et al., 2016), for microtubule end-binding proteins in cell lines (Smal et al., 2010) and synapses in brain tissue (Herold et al., 2010). However, in dense cultures, manual assignment of true synapses is not trivial and may suffer from inter-observer bias (Schmitz et al., 2011) (**Figure 4C**). Therefore, in absolute numbers, spot detection may also differ between ground truth and algorithm, but if counts change proportionally between low and high-density regions, the algorithm can at least be used to make relative comparisons.

To validate the pre- and postsynaptic colocalization measurements, controls need to be included that rely on staining for markers that should or should not colocalize (**Figure 4D**). For example, the combination of an inhibitory and excitatory marker (e.g., vGAT resp. AMPA-R) is not expected to colocalize and provides an indication of the non-specific, background detection level resulting from random colocalization. Two different primary antibodies targeting the same marker (e.g., synaptophysin) in turn, should provide an absolute value for the maximal number of colocalizing spots that can be detected. The combination of a pan-presynaptic marker (e.g., synaptophysin) and a specific postsynaptic marker (e.g., AMPA-R) should in turn yield an intermediate level of colocalizing spots, depending on the ratio of excitatory/inhibitory synapses. This strategy allows defining the dynamic range of the colocalization analysis.

Finally, key to being able to use synapse density as a reliable readout is the validation that it reports on changes in synaptic connectivity. This requires positive and negative controls, such as targeted modulation of synaptic protein levels by overexpression (as shown in **Figure 3B**) or shRNA-mediated knockdown. shRNA-mediated knockdown of PSD-95, gephyrin and synapsin has been reported to be effective for validation of synapse quantification (Nieland et al., 2014). However, reduction of essential synaptic components is bound to alter neuronal physiology and connectivity. Since synaptic connectivity is known to increase during maturation (Brewer et al., 2009; Verstraelen et al., 2014), cultures of different ages can also be used to validate the assay (**Figure 4E**). The density of colocalizing pre- and postsynaptic spots, i.e., mature synapses, is typically lower than the density of spots for the individual markers (Schätzle et al., 2012), depending on the nature of the marker used (turnover, target, and extrasynaptic presence) and definition for identifying synapses (proximity or overlap of pre- and postsynaptic spots).

During growth *in vitro*, not only the density of synapses increases progressively, but also the overall density of the network (Harrill et al., 2010; Verstraelen et al., 2014). This may complicate quantification of synaptic density in aged cultures. Ideally, chemical or genetic treatments are included that modulate the level of synaptic connectivity independent of the neurite network. Yet, many compounds that impair synaptic connectivity also affect parameters such as nuclei number, neurite density, and individual marker intensity, pointing to a general toxicity rather than a specific modulation of synapse numbers (Radio and Mundy, 2008; Harrill et al., 2011). As yet, few (if any) treatments have been identified that truly induce a robust increase in synapse density in otherwise unperturbed cultures. It can be anticipated that such a positive treatment will surface more quickly in cells grown under suboptimal conditions or in *in vitro* disease models with impaired synaptic connectivity.

### Synapse Density Screening in Practice

A few synapse screens have been reported in literature that were set up to identify novel synaptogenic factors. Proteins that induced a reduction in the synapse density after knockdown were considered synaptogenic. LRP6, a coreceptor for canonical Wnt ligands, was identified as a synaptogenic factor (Sharma et al., 2013). 3200 shRNA’s targeting 800 proteins were tested on a fully automated high-content screening platform using primary hippocampal neurons. LRP6 knockdown induced a 50% reduction in vGLUT1 + PSD-95 density. Subsequent analyses showed an equal reduction in dendritic spine density, but no alterations in vGAT + gephyrin clusters, showing that LRP6 is implicated in excitatory but not inhibitory synapse formation. In an analogous screening approach, Grin2C, a NMDA-R subunit, and axin-1, also part of the Wnt pathway, were identified as regulators of synaptogenesis (Nieland et al., 2014). Lastly, an RNAi screen on primary hippocampal neurons discriminated excitatory from inhibitory synapses by analyzing the overlap between PSD-95 + synapsin on the one hand, and vGAT + GABA-R on the other hand (Paradis et al., 2007). Knockdown of cadherins 11 and 13, important proteins for calcium-dependent cell–cell adhesion, resulted in decreased excitatory and to a lesser extent inhibitory synapse density. Subsequent electrophysiological analysis showed decreased functionality of excitatory synapses. This was also the case upon rem2 knockdown, a regulator of calcium channel assembly and trafficking. Furthermore, semaphorin 4D knockdown, a protein known to be involved in axon guidance, resulted in decreased inhibitory but not excitatory synapses. Thus, all of the reported synapse screens have made use of pre- and postsynaptic marker colocalization and have identified several proteins that regulate synaptogenesis.

## Dendritic Spines as Morphological Correlates of Excitatory Synapses

Throughout the continuum of spine shapes, different morphological stages such as filopodia, thin, stubby and mushroom spines can be distinguished. These shapes, and in particular the diameter of the spine neck, are believed to regulate the level of electrical and biochemical compartmentalization of the synapse (Adrian et al., 2014; Tonnesen et al., 2014). Spines appear, disappear or undergo morphological changes in response to learning paradigms by rapid rearrangement of the actin cytoskeleton, followed by a consolidation phase that is protein synthesis-dependent (Bosch et al., 2014). The link between spine density/morphology and synaptic strength makes spine-based assays popular in the study of synaptic connectivity (Kellner et al., 2014; Curto et al., 2016; Speranza et al., 2017). Yet, it must be noted that the structure-function relationship and its connection to memory formation is not fully elucidated and still subject of debate (Tonnesen et al., 2014; Segal, 2017).

Nonetheless, alterations in spine density and morphology have been documented in many neurodegenerative disorders (Herms and Dorostkar, 2016), as well as in neurodevelopmental diseases such as schizophrenia (Moyer et al., 2015) and autism spectrum disorders (Martínez-Cerdeño, 2017). Hypoxia was found to induce spine regression and the induction of filopodia (Segura et al., 2016). Conversely, 17β-estradiol treatment induced the formation of new spines and the recruitment of PSD-95, neuroligin-1 and the NMDA-R subunit GluN1 in those spines (Sellers et al., 2015). The application of normal BDNF, but not BDNF containing the val-66-met polymorphism that is carried by 30% of the world’s population, was shown to increase spine density and volume in primary hippocampal neurons (Xu et al., 2017). Dendritic spine abnormalities have also been replicated in *in vitro* models of mental disorders. Knockdown of the DISC1 gene in cortical neurons resulted in a 50% reduction in spine density after NMDA-R activation, while this activation did not elicit spine loss in control cultures (Hayashi-Takagi et al., 2014). This effect was abrogated by novel chemical inhibitors of p21-activated kinases. Likewise, *in vitro* models for neurodegeneration recapitulate spine loss, e.g., after mutated APP overexpression (Umeda et al., 2015) or application of β-amyloid oligomers (Jin and Selkoe, 2015; Freund et al., 2016).

Dendritic spines are present in primary neuronal cultures and can be resolved using high-resolution confocal microscopy (Papa et al., 1995; Boyer et al., 1998). However, most of the aforementioned published findings rely on manual analyses. Although by now, spine quantification can be done semi-automatically, to our knowledge, no large-scale high-content screens have been performed using this readout. This is mainly due to the stochastic labeling procedure (see below) and high image quality requirements, reducing the number of image material that can be used for analysis to a negligible quantity. Rendering spine quantification amenable to high-content screening demands a combination of targeted labeling and selective imaging strategies. Here below, we describe the existing spine analysis methods and we elaborate on how to render them more targeted.

### Resolving Dendritic Spines in Dense Neuronal Networks

**FIGURE 5:**
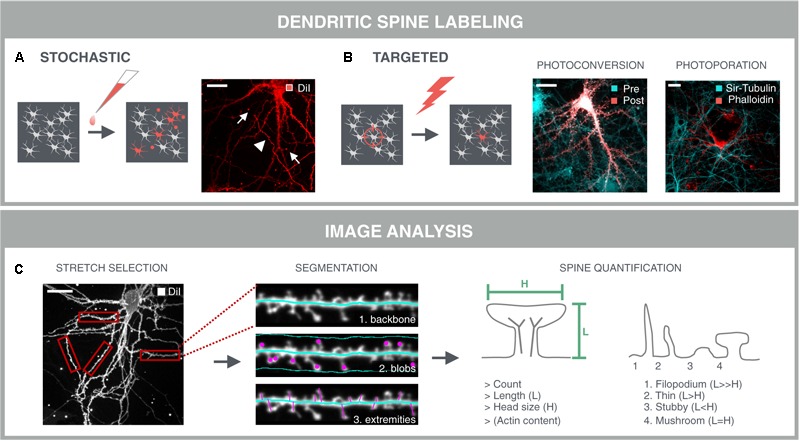
Dendritic spine analysis. Spine analysis requires sparse labeling of neurons and their spines in dense neuronal networks. **(A)** The gold standard method for spine labeling in culture is bath application of the lipophilic dye DiI. However, this method is not optimal for high-content screening due to the stochastic labeling of nearby neurites (arrowheads) and artifacts such as uneven dye loading and staining debris (arrows) which complicate subsequent image analysis. **(B)** Targeted labeling followed by imaging at the same locations drastically reduces the image acquisition time, as well as data storage and –analysis. Targeted labeling can be achieved with expression of a photoconvertible protein (mEos-LifeAct; Pre) that can be selectively converted (Post) in sufficiently separated neurons in the network. Alternatively, fast targeted labeling can be achieved by gold-nanoparticle (AuNP)-sensitized photoporation (Xiong et al., 2017, 2018). A single nanosecond light pulse heats up membrane-bound AuNP and thereby induces mechanical perturbation of the membrane, allowing otherwise impermeable AlexaFluor488-phalloidin to enter the neuron. The network is counter-stained with the cell-permeable probe Sir-Tubulin. **(C)** A first step for spine analysis involves the selection of analyzable dendrite stretches. This was done manually in this example and represents a current challenge for automated spine analysis. Segmentation of the dendritic backbone by skeletonization is either followed by blob detection in the vicinity of the dendrite, or by detection of the perpendicular extremities, i.e., spines, or by the combination of both methods. Several parameters are extracted such as spine count, length and diameter of the spine head. In case of mEos-LifeAct or phalloidin labeling, the actin content can be determined as well. Crude predictions of spine classes can be made based on their length and head size.

A prerequisite for dendritic spine analysis is a selective labeling of neurons and their spines that can be applied in a sparse manner. Labeled cells should be sufficiently separated from each other to avoid fluorescence from nearby neurites from masking that of spines. The gold standard for spine labeling is the lipophilic dye DiI (**Figure 5A**) (Cheng et al., 2014). This bright and photostable dye allows sparse labeling in culture by simple bath application. However, due to its stochastic nature, DiI staining often results in clustered staining of neuronal cells instead of isolated neurons, and it also labels non-neuronal cells. Moreover, artifacts such as uneven dye loading or staining debris complicate subsequent image analysis. In addition to fluorescent dyes, genetic labels such as cytoplasmic or actin-binding fluorescent proteins have been used in literature (Srivastava et al., 2011; Yang et al., 2015). Since actin is enriched in dendritic spines (Fischer et al., 1998), they offer a more selective target for labeling spines. However, sparse transfections always carry the risk of overexpression artifacts and, similar to DiI, they do not offer spatiotemporal control over the labeling. One way to make the labeling more targeted, is to make use of a photoconvertible protein, such as mEos-LifeAct (Paez-Segala et al., 2015). The conversion from green to red emission can be spatially controlled by directing the 405 nm photoconversion beam to sufficiently separated neuronal cell bodies within the dense network. Although this approach results in selective labeling of neurons and their spines, it is a slow (>1 h) procedure because multiple conversion pulses are required. Therefore, we have recently developed an alternative technique to rapidly label a multitude of selected cells, based on AuNP-enhanced photoporation (Xiong et al., 2017). In brief, the approach relies on illuminating neuronal cultures with a pulsed laser in the presence of membrane-tethered AuNPs and the fluorescently labeled actin-binding drug phalloidin. Localized heating around the AuNPs leads to the formation of vapor nanobubbles that transiently permeabilize the plasma membrane and allow the dye to enter the cell. Since the laser pulse only takes 7 nanoseconds, since multiple cells can be targeted in one shot, and since the illumination is guided through image content (Xiong et al., 2018), the technique is exquisitely suited for high-content purposes (**Figure 5B**). The additional advantage of having a targeted labeling strategy, is prior knowledge about the location of the labeled cells. In other words, targeted labeling directly implies targeted acquisition, and therefore increases the efficiency of the image acquisition.

### Automated Spine Quantification Requires Optimization of Image Analysis Routines

The accuracy of spine analysis critically depends on the image quality. In primary culture, spine lengths vary between 0.5 and 4 μm, and spine head diameters between 0.3 and 0.7 μm (Papa et al., 1995). Therefore, detailed morphological or volume measurements are difficult to perform when using conventional microscopy with a maximal lateral resolution of ∼0.2 μm and an axial resolution of ∼0.5 μm (Fouquet et al., 2015). Super-resolution modalities do allow more refined analyses, but are not yet set for high-throughput applications. Although several image analysis packages have been developed for 3D analysis of spines in fixed samples (Koh et al., 2002; Weaver et al., 2004; Cheng et al., 2007; Rodriguez et al., 2008), considering the three times lower axial resolution, a simpler 2D analysis may suffice for screening purposes. Such pragmatic approach yields more rudimentary readouts such as spine number, length and head diameter that can be used for crude predictions of spine classes.

When using an actin-binding fluorescent marker such as mEos-LifeAct or phalloidin, a very rudimentary but fast option would be to quantify the actin content by simple spot segmentation. However, this approach favors the detection of spines with large heads and could leave other spine types undetected. Moreover, robust assessment of spine density demands a unit to normalize to, usually a dendritic stretch. Therefore, most approaches rely on detection and skeletonization of the dendritic backbone, followed by the identification of spines either as blobs within a confined region along the backbone, or as perpendicular extremities of the dendritic backbone (**Figure 5C**). The former is often used for the detection of detached spines, i.e., spines lacking a detectable neck, while the latter only detects attached spines (Koh et al., 2002; Weaver et al., 2004; Cheng et al., 2007). Another approach consists in detecting the outer tips of potential spines by adaptive local thresholding, followed by voxel clustering to trace back the spine tip to the dendritic backbone (Rodriguez et al., 2008). Providing a detailed description of these algorithms is not the primary aim of this review [for a more detailed review, see (Detrez et al., 2016)], but all packages use isolated dendrite stretches as input and often require tailored analysis settings for different samples. Thus, even with targeted labeling and imaging strategies, parts of the image cannot be analyzed (crossing neurites, soma) and should be discarded. Moreover, several studies have reported differential spine characteristics on proximal and distal dendrites (Weber et al., 2016; Walker et al., 2017), calling for a cell location-specific assessment. In low-throughput mode, analyzable segments can be selected manually (**Figure 5C**), but for screening, selection and localization of dendritic segments should be automated. To this end, one could consider building a library of reliable stretches and use machine learning to recognize analyzable dendrites during high-content screening. Although relatively new in the field of neuroscience, machine learning approaches have already been used for tracing of neurites (Gala et al., 2014), and to classify cortical neurons in histological sections according to their morphology (Vasques et al., 2016). Recently, a machine learning approach was also used for spine classification, which outperformed morphological feature-based methods (Ghani et al., 2017).

## Measuring Molecular Fluxes at the Synapse

The presence of synapses is a strong indicator of connectivity, but it does not necessarily imply that these connections are functionally active. A desired goal is to monitor the electrical signals generated by many neurons simultaneously, as well as the information transfer at the synaptic terminals (Miesenbock et al., 1998). Furthermore, deficiencies in synaptic function have been implicated in many neurological and psychiatric diseases (Forero et al., 2006; Mandolesi et al., 2015; Henstridge et al., 2016; Lepeta et al., 2016; Keller et al., 2017). The classical technique employed for this purpose is electrophysiology, but despite its strong merits, it is less suitable for high-throughput drug screening assays [although advancements are being made (Obergrussberger et al., 2015)].

### Fluorescent Reporters of Synapse Activity in Live Neuronal Networks

A scalable imaging-based approach has come to the fore, where fluorescent reporters can be used to report different aspects of cellular activity. The routinely used small molecule dyes such as Fluo-4 and OGB1 are used to measure changes in Ca^2+^ concentration, di-8-ANEPPS (Beach et al., 1996) or Annine-6 (Kuhn et al., 2004) for imaging membrane potential, and FM1-43 to track exocytosis, endocytosis, and recycling of secretory granules or vesicles (Betz and Bewick, 1992). Although very useful, they have their own sets of limitations that restrict their applicability: (1) the bulk dye loading procedures such as the AM esters, label all the cells indiscriminately and therefore, it is difficult to obtain cell-type specific information; (2) because these dyes label the entire cell, it is rather difficult to identify neuronal compartments – particularly in a high-throughput setting; (3) they have a very short utility window lasting only for a few hours typically, which prevents longitudinal imaging of neuronal activity. Therefore, genetically encoded fluorescent reporters of neuronal activity are becoming the tools of choice. It should be noted that the use of these fusion proteins may elicit similar overexpression (or chelating) artifacts as discussed for fixed samples in section “Visualizing Individual Synapses”. However, the problems associated with overexpression can be mitigated by using inducible reporter systems so that the window of protein expression is kept to a minimum (Atze et al., 2016).

A variety of GECIs such as the GCaMP family (Nakai et al., 2001; Chen et al., 2013) and red-shifted variants such as the RCaMP and R-GECO family (Zhao Y. et al., 2011; Dana et al., 2016) have been developed, offering flexibility in terms of timing (e.g., long-term, repetitive follow-up), cell-type specificity (e.g., exclusive neuronal expression via the hSyn1 promoter) and localization. At the neuronal level, intracellular calcium fluxes are considered to represent the downstream molecular consequence of electrical depolarization due to action potential firing (Smetters et al., 1999; Herzog et al., 2011). Hereby, an increased synchronicity between cells of a cultured network is interpreted as a higher degree of functional synaptic connectivity (Verstraelen et al., 2014). However, alternative stimuli, such as activation of extrasynaptic NMDA receptors (Vanhoutte and Bading, 2003) and astrocytic signaling (Pál, 2015) may contribute to intracellular calcium dynamics as well, and a calcium signal that is measured in the soma of a neuron is the result of an interplay between several hundreds of synapses and summation of action potentials. A more resolved view can be obtained by targeting GECI to pre- or post-synaptic terminals. This can be achieved by fusing them with either synaptophysin or PSD-95, respectively (Dreosti et al., 2009; Hempel et al., 2011; Reese and Kavalali, 2016). Because the calcium reporter is now localized at the terminal, one can identify locations of synapses active in the circuit, which is useful for relating circuit function to structure. Similarly, information about vesicle exocytosis and endocytosis can be obtained using probes such as SypHy or sypHTomato (Granseth et al., 2006; Li and Tsien, 2012), and the release of the major neurotransmitter glutamate can be robustly quantified using the intensity-based Glutamate Sensing Fluorescent Reporter, iGluSnFR (Marvin et al., 2013). A comprehensive overview of state-of-the-art reporters is beyond the scope of our review but the interested reader is directed to these excellent reviews (Dreosti and Lagnado, 2011; Broussard et al., 2014; Lin and Schnitzer, 2016; Rodriguez et al., 2017; Sepehri Rad et al., 2017). Moreover, using spectral multiplexing – that is by measuring responses from two spectrally distinct reporters at the same time – multiple aspects of synapse functionality can be investigated simultaneously.

### Microscopic Imaging of Spontaneous and Evoked Synaptic Activity

Low-throughput methods for the culture and transduction of primary hippocampal neurons, imaging, and analysis of fluorescence signals down to the resolution of individual synaptic vesicles are well documented (Burrone et al., 2007; Royle et al., 2008; Zhao C. et al., 2011). However, upscaling is non-trivial, mostly because live cell imaging experiments put a higher demand on the imaging modalities: the microscope needs environmental control (37°C, 5% CO_2_, 100% relative humidity) and measures to prevent focus drift (continuous focus correction, anti-vibration table). For fixed samples, only photobleaching, i.e., irreversible de-activation of fluorochromes under the influence of light, poses a potential problem. However, for live cells also the production of ROS, dimerization of DNA base pairs, and local heating, collectively termed phototoxicity, come into play (Magidson and Khodjakov, 2013). Photobleaching and phototoxicity can be mitigated by limiting the excitation light, yet this occurs at the expense of temporal sampling frequency, total observation time and/or SNR. As signal fluctuations should be captured over time with a sufficiently high temporal resolution, across a large number of synapses, and this for large populations of cells, the imaging setup requires a sensitive detection and a large field of view. Due to the additional time dimension, the information content of live cell recordings is much richer, putting an additional demand on data storage and analysis.

When considering calcium imaging, neurons in a culture typically show some degree of spontaneous firing behavior, depending on their maturity (Verstraelen et al., 2014). However, to assay changes in synaptic transmission upon pharmacological treatments, activity is often evoked, using electrical or optogenetic stimulation (Wagenaar et al., 2004; Barral and Reyes, 2017; Zhang and Cohen, 2017). The delivery of stimuli represents an additional level of complexity for the imaging setup, especially for electrical stimuli since electrodes and stimulation hardware should be in place. Typically, the stimulus given to the neuronal culture is calibrated; for instance, just enough depolarization to induce a single action potential (Wardill et al., 2013). The fact that the stimulus can be precisely controlled also allows for the construction of more refined experiments regarding neuronal network response to, e.g., fast and slow stimuli, or to more complex stimuli patterns mimicking different types of sensory input. As through the use of optogenetic stimulation and a digital micro-mirror device, spatiotemporal patterns can be induced as well (Zhu et al., 2012). The major advantage of active stimulation is a direct causality and synchronization of the response. A potential drawback is that the type of response may differ, since it has been shown that spontaneous and evoked transmission is driven by different sets of synapses (Peled et al., 2014), which respond in a different way to chemical perturbations (Reese and Kavalali, 2016).

### Adding Throughput to Functional Imaging

When aiming to identify compounds that modulate synaptic plasticity, a screening system that is robust and has a decent throughput is required. In that context, a neuronal screening system needs to comprise culturing and imaging of hippocampal neurons in 96-well plates, automated analysis, and storage and management of the generated data. A leading example of such a system is MANTRA (Hempel et al., 2011), which allows high-throughput fluorescence acquisition of neurons/synapses while they are subjected to field electrical stimulation. However, a major limitation of this system is that it collects fluorescence from all the 96 wells at the same time using a 96-minilens array. Although this increases the speed of acquisition, the acquired signal is an average of the entire well space, i.e., the signal is averaged over the actual synapses, non-specifically labeled puncta, and blank space. This “smeared” signal prevents the detection of spatiotemporally defined events and provides no view on the stochasticity of synaptic transmission. More recently, a system was developed that combines imaging at higher resolution with electrical field stimulation (Wardill et al., 2013). Although it was developed as a screening system for optimizing GECIs, such an imaging approach may very well be amenable to study the effect of compounds on the synaptic activity in cell cultures. A similar approach was adopted by Virdee et al. (2017) who combined spatially confined electrical field stimulation with calcium imaging at a distant position in the well. The calcium response, elicited at one position and recorded at another, was attenuated by an AMPA-R antagonist, indicating that the electrical stimulus was transmitted to other neurons in the well via AMPA-R-containing synapses.

### Analyzing Synaptic Signals Across Time

**FIGURE 6:**
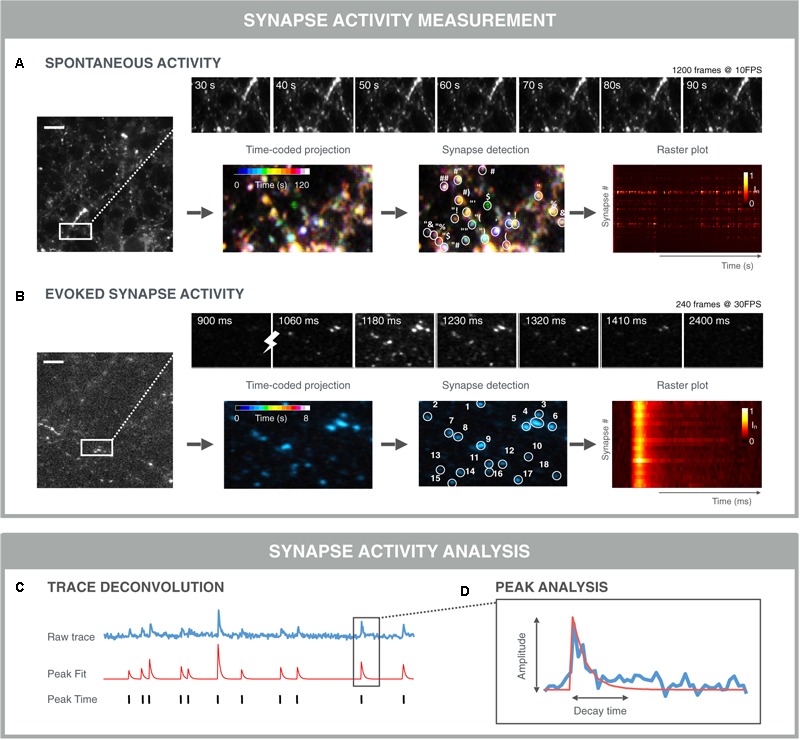
Examples of functional synapse analysis. **(A)** Time-averaged intensity of a 120 s SyGCaMP6f fluorescence microscopy recording of spontaneous presynaptic activity as measured. Top: montage of selected time points showing fluctuations in individual synapses. Bottom: color-coded time projection with dissimilar colored synapses indicating non-simultaneous activity; synapse detection is based on spatial and/or temporal features; the raster plot shows the normalized intensity of detected synapses over time. Both synchronous and asynchronous signals are visible between traces of different synapses. **(B)** Time-averaged intensity of 8 s Synaptophysin-GCaMP6f (SyGCaMP6f) fluorescence recording of evoked responses measured at the presynaptic terminals. The stimulation was 1 AP. Top: montage of selected time points showing synchronous responses to stimulation (lightning bolt). Bottom: Time-coded projection: due to the stimulation and thereby synchronous activation, synapses are similarly colored; synapse detection algorithms can exploit the stimulus properties in addition to the spatio-temporal synapse response features, which can result in more robust segmentation. Downstream signal analysis involves **(C)** trace deconvolution and **(D)** peak analysis. Typically, an exponential curve is fitted with amplitude and decay time.

Automation of image acquisition also demands automation of downstream image analysis (**Figure 6**). Typically, in morphological assays, synapses are detected as Gaussian blobs (see section “Quantification of Synapse Density”). However, in functional assays, not all synapses are active at all times and thus the detection of individual synapses in a single time point becomes more difficult. Yet, it is exactly the time dimension that can be used to aid with the detection of active synapses. The simplest approach would be to project the time stack (e.g., by taking the average intensity of each pixel position across time), and subsequently apply a typical blob detector (Cormen et al., 2001). In dense cultures, this approach is not ideal since it leads to the merging of signals from nearby synapses. More advanced detection methods determine correlated signal fluctuations to define whether adjacent pixels belong to the same synapse (Portugues et al., 2014; Pnevmatikakis et al., 2016). Such an approach can be refined further by including constraints and generative models for synapses, background and noise signals (Pnevmatikakis et al., 2016). A downside of this approach, is that it typically results in a very large constraint optimization problem which is computationally demanding. To address this, a solution was recently proposed which analyzes smaller time windows progressively (Giovannucci et al., 2017).

Once synapses are detected, signals can be analyzed per synapse. For spontaneous fluctuations, the analyses are more challenging than for evoked fluctuations, as the responses are usually less pronounced and more stochastic (**Figure 6A**). In such a case, a peak finding algorithm can be applied to infer the timing of a signal increase. Again, a wide range of algorithms are being used ranging from simple deconvolution, over Bayesian inference, template matching to convolutional neuronal networks (Vogelstein et al., 2009; Pnevmatikakis et al., 2016; Friedrich et al., 2017). A challenge with comparing these methods is that it is difficult to construct good benchmarking data sets, since artificial sets do not contain the same noise and features of real data. Additionally, for real data, the true spiking behavior is not always known (Pachitariu et al., 2017; Berens et al., 2018). Nevertheless, spike deconvolution algorithms are becoming more mature and robust, allowing for a substantial computational increase in resolution. When peaks are evoked, the timing of the stimulation is known and extraction of peak responses can be performed more easily and precisely (**Figure 6B**). When peaks are extracted, individual peaks can be modeled and characterized in terms of amplitude, rise- and decay time (Gerstner et al., 2014). At the synapse level, additional metrics can be extracted such as the firing rate and peak variance (Moreaux and Laurent, 2008). Finally, at the population level, the relative timing of peaks across synapses can be measured to study signal propagation within, e.g., action potential propagation along the axon, or between neurons. In case of stimulated activity, such relative timing between synapses may inform about the propagation of this stimulus from its initial position to connected neurons throughout the network. For spontaneous activity, the apparently synchronized network events may be traced back to a single neuron or to a group of neurons that trigger the network activity. Such refined methods may uncover subtle differences in network connectivity during treatment or may accelerate the detection of in *in vitro* disease states that are less detectable at the level of the individual synapse.

## Future Perspective

### Toward Integrative Synapse Screening

Several readouts for synaptic connectivity have been described in the literature, but, as of yet, large scale high-content screens that aim to identify novel pathways involved in synapse plasticity remain scarce. Of the three readouts discussed above, the quantification of pre- and postsynaptic markers and their colocalization is currently the most feasible for high-content screening (Paradis et al., 2007; Sharma et al., 2013; Nieland et al., 2014), provided that the labeling, imaging and image analysis procedures are thoroughly validated. Despite the proven value of the standard synapse density screen in identifying novel synaptogenic factors, its variability and limited dynamic range may mask the effects of more subtle modulators. This is predominantly due to the variable growth and density of neurons, especially in aged (>14 DIV) cultures. Thus, one way to boost the sensitivity could be to grow neurons on micropatterned substrates in order to gain more control over the growth pattern of synapses and avoid fasciculation of neurites, which undermines accurate synapse quantification (Czondor et al., 2013; Burbulla et al., 2016). Alternative approaches to investigate synapse formation in a more targeted manner consist of co-culturing neurons with non-neuronal cells that overexpress synapse-attracting proteins on their plasma membrane. This has led to the identification of a series of synaptogenic proteins including the leucine-rich repeat transmembrane (LRRTM) (Linhoff et al., 2009). The combination of both approaches has also been reported: by positioning neuroligin-1-expressing HEK293 cells in regularly spaced microwells and guiding axonal growth in microchannels toward the HEK293 cells, synapses were spatially confined (Shi et al., 2011). Consequent quantification of synaptic ‘patches’ allowed the identification of histone deacetylase inhibitors as promoters of synaptogenesis. Although fast and sensitive, the scope of such an advanced system is limited compared to the ‘standard’ synapse assay described in section “Visualizing Individual Synapses,” since it only allows for the identification of treatments that influence the attraction of presynaptic terminals by a single postsynaptic membrane protein, in this case neuroligin-1.

As opposed to synapse screens, no reports have been published so far on high-content analyses of spine density and morphology in neuronal cultures. Currently, there are two unmet criteria for upscaling: reproducible fluorescent labeling and robust, user-independent image analysis. Targeted labeling methods – as offered by spatiotemporally controlled techniques such as photoporation – will significantly contribute to upscaling. Intelligent imaging, i.e., high-resolution imaging being guided by prior analysis of low-resolution images (Russel et al., 2010), in turn is bound to raise the efficiency of both labeling and imaging procedures. But, even with targeted labeling and imaging, selective image analyses that rely on machine learning, will be needed to recognize analyzable regions.

Although their value has been proven in several literature reports (see sections “Synapse Density Screening in Practice” and “Dendritic Spines as Morphological Correlates of Excitatory Synapses”), the presence of morphological correlates such as spines does not necessarily report on synapse function. Direct comparisons of morphological readouts with network functionality are scarce, but those available often describe discrepancies between morphology and function. While the expression of synaptic proteins has been shown to increase linearly with culture age, the network’s activity scales exponentially, showing that synapse formation drastically increases the information processing capability of the network (Brewer et al., 2009). Enhanced trophic support by an astrocyte feeder layer or astrocyte-conditioned medium was shown to increase dendritic spine density and to a lesser extent synchronous calcium activity, while neurite- and synapse marker density remained unchanged. Interestingly, reducing such support by deprivation of NGF did not induce the opposite effects, as synchronous bursting was severely affected, while network morphology (neurites, synapse markers and spines) was unaltered (Verstraelen et al., 2014). Microtubule hyperstabilization by overexpression of human Tau impaired synchronous calcium bursting and decreased neurite density, while the synapse number remained unchanged (resulting in an increased synapse density due to the neurite retraction) (Verstraelen et al., 2017). These discrepancies show that a combined interpretation of both morphological and functional assays is needed to truly grasp the effect of experimental perturbations. Hence, the logical next step would be to integrate the combined feature sets using supervised or unsupervised machine learning techniques to identify subtle changes in synaptic connectivity and to predict the mode of action of compounds with unknown function. Importantly, such comprehensive profiling of synaptic connectivity requires significant storage capacity, computational power and a solid theoretical framework. Standardized data analysis strategies have been conceived to objectively categorize and visualize multidimensional data from high-content screens (Grys et al., 2016; Caicedo et al., 2017). Computational models are being optimized to aid with the biological interpretation but also to benchmark new image analysis algorithms, and this in turn is supported by improved simulations of cellular models and networks (Loew and Schaff, 2001; Lenk et al., 2016). Finally, the models, images and morphofunctional data should be bundled and centralized to make them openly available to the neuroscience community, as was recently done for neuronal morphology (Parekh and Ascoli, 2013; Akram et al., 2018).

### Next-Generation Microscopy Paradigms

Microscopy technology is evolving at a rapid pace. Super-resolution imaging has provided unprecedented insights into the organization of synaptic proteins and the postsynaptic density, but as of yet only at a low throughput (Izeddin et al., 2011; MacGillavry and Hoogenraad, 2015; Lee et al., 2017). Recently, however, universal point accumulation imaging in nanoscale topography (uPAINT) has been proposed as a novel method for rapid acquisition of super-resolved images on a standard wide-field microscope (Giannone et al., 2013). This technique relies on the recording of high numbers of single molecules at the surface of a cell by constantly labeling while imaging. Another high-content setup for single-molecule localization microscopy relies on simultaneous acquisition and processing of images, combined with advanced data mining to increase the throughput (Beghin et al., 2017). Not only the spatial, but also the spectral resolution can be extended. By repetitively staining, imaging and photobleaching, automated imaging cycler microscopy allows the imaging of hundreds of proteins in the same sample (Schubert, 2015). This fully automated technique could allow the unraveling of the synaptic toponome and the identification of protein networks responsible for CNS pathology. Finally, the time resolution is continuously being improved. Indeed, it is now feasible to directly monitor changes in membrane potential at high temporal resolution using voltage-sensing optical probes (Borden et al., 2017). Combined with optogenetic stimulation, all-optical electrophysiology studies have been conducted to study the function and pharmacology of voltage-gated ion channels in cells (Zhang et al., 2016), as well as neuronal activity in ganglia and brain slices derived from transgenic Cre-dependent ‘Optopatch’ mice (Lou et al., 2016). Though voltage imaging has been used in high-content screens aimed at identifying novel potassium channel inhibitors in non-neuronal cell lines (Solly et al., 2008), no screens have been reported in neurons, plausibly because the faster kinetics of action potentials. Yet, with the rapid evolution of markers, microscopy technology and automated image analyses, high-throughput interrogation of synaptic connectivity in neuronal culture is becoming the mainstay in target identification/validation and phenotypic drug screening.

### Boosting the Translational Value

This review focused on primary neuronal cultures obtained from mouse or rat to model synaptic connectivity *in vitro*. Yet, the analyses equally apply to neuronal cells of human origin (Pruunsild et al., 2017). Synaptic markers and synchronous network activity have already been quantified at low throughput in human iPSC-derived neuronal networks (Nieweg et al., 2015; Kuijlaars et al., 2016; Nageshappa et al., 2016). In contrast, dendritic spines have only been observed upon differentiation and integration in organotypic slice cultures (Hiragi et al., 2017; Miskinyte et al., 2017). Recently, neuronal differentiation protocols have become much faster (Mertens et al., 2016). Currently, the fastest protocols yield electrically active neurons after 3–4 weeks, by direct reprogramming of fibroblasts into neurons, thereby bypassing the progenitor stage (Hu et al., 2015; Miskinyte et al., 2017). Further optimization of differentiation protocols in terms of speed and reproducibility, as well as thorough characterization of the obtained neuronal networks, may render high-content screening feasible in the future. It is important to note that neurons do not grow as isolated cells, but also require additional cell types such as astrocytes for their proper functioning. Astrocytes ensheath synaptic terminals [hence, the term tripartite synapse (Alberto and Alfonso, 2013)] and are critical for initial synapse formation, maintenance and synaptic transmission (Barres, 2008; Panatier et al., 2011; Dallérac et al., 2013). Furthermore, as all neuropathologies show reactive astrocytosis, loss of synapses and neurons in Alzheimer’s disease may well be triggered by astrocyte dysfunction. Therefore, future work needs to focus on mimicking the physiology and function of astrocytes in neuronal culture systems. But even in such co-cultures, not all types of brain cells are present, the 3D context is missing, and neurons form random connections with each other, in contrast to the layered organization of cortex and hippocampus. More complex *in vitro* models that are gaining attention, are human brain organoids (Lancaster et al., 2013). These self-organizing 3D models develop a layered structure and contain all cell types of a real brain (Quadrato et al., 2017). For high-content screening, such organoids demand novel imaging approaches such as light sheet microscopy combined with intelligent sample mounting (Pampaloni et al., 2015). A more advanced approach consists of combining fluidics for flow cytometry with light sheet imaging (Gualda et al., 2017). In this setup, organoids travel at a constant speed through a light sheet to acquire several optical sections with subcellular resolution. The throughput of such a system is determined by the fluorescence intensity and camera speed. As such, high-content screening may eventually become feasible, e.g., in the context of neurodevelopmental disorders where cerebral organoids can be analyzed in an immature state, e.g., after 1 month (Zhou et al., 2017).

In conclusion, owing to its high plasticity, the synapse is an extremely informative biomarker of neuronal (dys-)function. High-throughput microscopy now allows extraction of quantitative information on synapse density, composition and function with statistical power. The integration of the different readouts will offer a more comprehensive view on connectivity, while the development of physiologically more relevant models will boost the translational value of synapse screens. Eventually, this should amount into an expedited development of novel therapies against neurodevelopmental and neurodegenerative disorders that currently affect not only a large number of patients, but also their caregivers, and the global economy.

## Author Contributions

PV, NK, RN, and WDV conceived the work. MVD and MV performed the image analysis and annotation. PV, MVD, NK, J-PT, and WDV drafted the manuscript. All authors critically revised the manuscript and approved it for publication.

## Conflict of Interest Statement

NK and RN are full time employees of the pharmaceutical company Janssen Pharmaceutica NV. The remaining authors declare that the research was conducted in the absence of any commercial or financial relationships that could be construed as a potential conflict of interest.

## Abbreviations

AMPA-R: α-amino-3-hydroxy-5-methyl-4-isoxazolepropionic acid receptor
APP: amyloid precursor protein
AuNP: gold nanoparticle
BDNF: brain-derived neurotrophic factor
DISC1: disrupted-in-schizophrenia-1
DIV: days *in vitro*
ENABLED: endogenous labeling via exon duplication
FingR: fibronectin intrabodies generated with mRNA display
GABA: γ-aminobutyric acid
GECI: genetically encoded calcium indicator
GFP: green fluorescent protein
iGluSnFR: intensity-based glutamate sensing fluorescent reporter
LRRTM: leucine-rich repeat transmembrane protein
MANTRA: multiwell automated neurotransmission assay
MAP2: microtubule-associated protein 2
NA: numerical aperture
NGF: nerve growth factor
NMDA-R: *N*-methyl-D-aspartate receptor
OGB1: oregon green-bapta-1
PSD-95: postsynaptic density protein-95
ROS: reactive oxygen species
SNR: signal-to-noise ratio
uPAINT: universal point accumulation imaging in nanoscale topography
vGAT: vesicular GABA transporter
vGLUT1: vesicular glutamate transporter 1
